# The Challenge of Periprosthetic Joint Infection Diagnosis: From Current Methods to Emerging Biomarkers

**DOI:** 10.3390/ijms24054320

**Published:** 2023-02-21

**Authors:** Corneliu Ovidiu Vrancianu, Bogdan Serban, Irina Gheorghe-Barbu, Ilda Czobor Barbu, Roxana Elena Cristian, Mariana Carmen Chifiriuc, Catalin Cirstoiu

**Affiliations:** 1 Microbiology Immunology Department, Faculty of Biology, University of Bucharest, 050095 Bucharest, Romania; 2 The Research Institute of the University of Bucharest, 050095 Bucharest, Romania; 3 University Emergency Hospital, Carol Davila University of Medicine and Pharmacy, 020021 Bucharest, Romania; 4 Department of Biochemistry and Molecular Biology, Faculty of Biology, University of Bucharest, 050095 Bucharest, Romania; 5 Romanian Academy, 010071 Bucharest, Romania

**Keywords:** periprosthetic, joint infection, arthroplasty, biomarkers, synovial culture

## Abstract

Due to the increase in the life span and mobility at older ages, the number of implanted prosthetic joints is constantly increasing. However, the number of periprosthetic joint infections (PJIs), one of the most severe complications after total joint arthroplasty, also shows an increasing trend. PJI has an incidence of 1–2% in the case of primary arthroplasties and up to 4% in the case of revision operations. The development of efficient protocols for managing periprosthetic infections can lead to the establishment of preventive measures and effective diagnostic methods based on the results obtained after the laboratory tests. In this review, we will briefly present the current methods used in PJI diagnosis and the current and emerging synovial biomarkers used for the prognosis, prophylaxis, and early diagnosis of periprosthetic infections. We will discuss treatment failure that may result from patient factors, microbiological factors, or factors related to errors during diagnosis.

## 1. Introduction

Due to the increase in life span and mobility at older ages, the number of prosthetic joint implants is constantly increasing [1]. Arthroplasty surgery is safe and effective in terms of symptom alleviation, recovery of functions, and quality of life, especially in the elderly. However, the increase in the number of prosthetic joint replacements also leads to increased postoperative complications [2]. Prosthetic joint infections (PJIs) are among the most severe complications that occur after total joint arthroplasty, having an incidence of 1–2% in the case of primary arthroplasties and up to 4% in the case of revision operations. Furthermore, they cause a huge economic burden on the health care system. A substantial increase in the number of patients diagnosed with PJIs is expected in the coming years due to the increasing volume of total joint arthroplasties performed internationally [3,4]. However, the optimization and development of efficient protocols for managing PJIs can lead to the establishment of preventive measures and the most effective diagnostic methods based on the results obtained after laboratory tests [5,6].

The long life of an implant is associated with an increased risk of infections. The implantation of a foreign body increases the pathogenicity of bacteria, and bacterial biofilms make the diagnosis complex and challenging. The most common microorganisms found in hip and knee replacement are *Staphylococcus aureus* and coagulase-negative staphylococci. In addition, pathogenic bacteria colonizing other parts of the body, such as the bacterium *Cutibacterium acnes*, are encountered in arthroplasty shoulder operations. In contrast, Gram-negative bacteria lead to infections that occur after hip replacement surgeries [7]. Depending on the microbial etiology and virulence of the involved strains, the PJI may occur early (in the first month after implantation), when highly virulent bacteria (*S. aureus*, streptococci, enterococci) are involved, or up to three years after implantation, when bacteria with lower virulence, such as coagulase-negative staphylococci and *Cutibacterium* species, are encountered [2].

Early diagnosis of PJI is essential in saving the prosthetic implant and joint function. However, many methods are insufficiently sensitive and specific for diagnosing PJI. Therefore, a combination of laboratory studies, microbiology, histopathology, imaging, and molecular studies is needed to provide an accurate diagnosis. This review will present the current methods used in PJI diagnosis (microbiological, histopathological, imaging, and molecular), as well as the current and emerging synovial biomarkers used for the prognosis, prophylaxis, and early diagnosis of periprosthetic infections. We will also discuss treatment failure that may be the consequence of patient and microbiological factors or factors related to errors during diagnosis.

## 2. PJI Diagnosis

**Figure 1:**
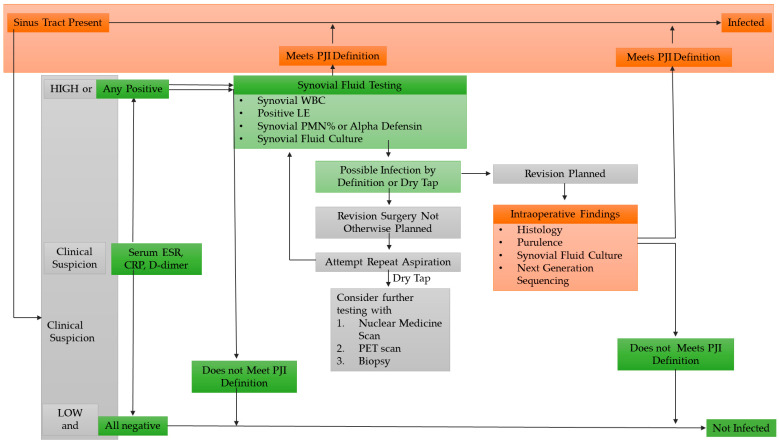
Criteria and strategies used in PJI (periprosthetic joint infection) diagnosis. Legend: ESR, erythrocyte sedimentation rate; CRP, C-reactive protein; WBC, white blood cells; LE, leukocyte esterase; PMN, polymorphonuclear neutrophil (modified from Alvand et al., 2020 [15] with permission).

Several criteria to be considered for establishing a correct diagnosis of PJIs have been published by the American Society of Infectious Diseases [8,9] (Figure 1). One of the criteria to be considered when making a diagnosis is the presence of clinical signs. The clinical signs of acute infection can be systemic, such as fever and local (pain, erythema, edema, and impaired joint function). However, if causes such as adverse reaction to the metal or reactive arthritis are excluded, one of the most evident signs confirming the infection is the presence of a sinus tract or a purulent around the prosthesis [10].

One of the most-used classifications of periprosthetic infections was suggested by Coventry (1), who proposed three distinct stages of the infection: the acute phase (in the first three months), phase 2 (more than three months after the operation), and phase 3 (at two years after infection) [11]. Another intensively cited classification is the one formulated by Tsukayama et al., who proposed a classification system for periprosthetic infections consisting of four groups: positive intra-operative cultures, early postoperative infection occurring before four weeks, late chronic infection (>4 weeks), and acute hematogenous infection [12]. The timing of intervention and the duration of the symptoms are considered the best prognostic factor in eradicating infection. For these reasons, Romano et al. proposed a seven-point PJI classification that focuses on several issues, including the responsible microorganisms, host status, bone, soft tissue defects, etiopathogenesis, and anatomical and pathological features, from acute with rapid-onset pain [13]. More recently, Pellegrini et al. proposed a new classification scheme that focuses on the identification of different characteristics of infection based on the topography of the infectious process. This theory is based on identifying the exact location of the bacterial colonization and guides the treating surgeons, allowing them to decide between a conservative or a more radical intervention irrespective of the timing [14]. These classification schemes can be used to guide clinicians in therapeutic decision making.

### 2.1. Microbiological Diagnosis of PJI

The etiological diagnosis is one of the most critical steps to establish a correct treatment that will lead to the best results [16]. The microbiological study can be performed both from synovial fluid samples collected before surgery, from tissue samples obtained during the operation, and by sonication to dislodge the adherent microorganisms from the prosthetic joint surface using the dithiothreitol (DTT) technique or by bead mill processing.

Kim et al. compared the results of a culture method using sonication with the conventional culture method for patients with an infected total knee arthroplasty. They found that a culturing workflow incorporating sonication diagnosed pathogens in patients with an infected total knee arthroplasty with higher sensitivity than the conventional culturing method [16].

Recently, van Schaik et al. conducted a systematic review to establish the clinical validity of intraoperative tissue cultures and their concordance with preoperative synovial fluid culture. The authors observed a concordance rate of 78% between preoperative synovial fluid cultures and intraoperative tissue cultures, meaning that in approximately one in three to four cases, the results of the intraoperative tissue cultures do not match with the earlier results of the preoperative synovial fluid culture. Consequently, when using the preoperative synovial culture as a single test to distinguish between septic or aseptic implant failure, one in three to four cases may be misdiagnosed and subsequently be under or over-treated. The results suggest that a clinician needs to confidently establish a postoperative treatment strategy based on the preoperative cultures alone [17].

A retrospective evaluation that investigated 35 patients showed that preoperative joint aspirations are likely to miss some bacterial species (*Cutibacterium* sp., coagulase-negative staphylococci). Still, this investigation is recommended to ensure the maximum efficiency of the targeted antibiotic therapy [18]. Furthermore, stopping antibiotic treatment for up to 14 days increases the sensitivity of the culture obtained by sonication [19].

Recently, novel approaches with chemical agents have been investigated for biofilm dislodgement, such as DTT. De Vecchi et al. evaluated the applicability of DTT treatment for processing periprosthetic and osteoarticular tissues for diagnosing bone and joint infections. They compared it with regular saline solution treatment. Concordance between the two methods was observed in 85.7% of 70 cases. Sensitivity was 88.0% for DTT and 72.0% for saline. Specificity was 97.8% and 91.1% for DTT and saline, respectively. Treatment with DTT showed higher sensitivity and specificity, suggesting that it may be considered a viable strategy for microbiological analysis of tissues to diagnose bone and joint infections [20].

More recently, Randau et al. evaluated the performance of a commercial DTT kit in 40 revision arthroplasty cases and obtained a sensitivity of 65% and a specificity of 100%, compared with conventional microbiological cultures [21]. Karbysheva et al. compared the biofilm dislodgement efficacy of DTT to the sonication procedure in diagnosing PJI in a prospective cohort including 187 patients undergoing hip and knee prostheses explantation. Sonication showed better sensitivity (73.8%) than DTT (43.2%) for the diagnosis of PJI and comparable specificity (98% and 94.6%, respectively), suggesting that sonication provides a more reliable diagnosis of PJI and detects approximately 30% more pathogens compared with DTT systems [22].

Another method used to treat PJI tissue samples is the bead mill process. In 2011, Roux et al. developed a semi-automated mechanized disruption technique for solid tissue samples to improve the microbiological PJI diagnosis. They submitted 495 samples collected from 92 consecutive patients with PJI revision surgery to bead mill processing. They used the bead milled suspension to seed solid and liquid media. As a result, they obtained the highest diagnosis rate (83.7%) compared with other techniques [23]. Two studies conducted by Warnke et al. and Redanz et al. demonstrated that bead mill tissue sample homogenization is superior to regular sample collecting and processing methods. In addition, they observed that the recovery of microorganisms is significantly higher than conventional processing methods [24,25].

Currently, the conventional method of synovial fluid and tissue sample cultures does not meet the conditions set by the doctors responsible for establishing a treatment. This is demonstrated by the high rate of false-negative and false-positive results [26]. Problems with PJI false-positive and negative results are associated with the presence of biofilm, which plays a central role in the pathogenesis of the infection. Current in vitro susceptibility tests fail to effectively assess the ability of antibiotics to kill bacteria embedded in a complex structure such as biofilm. To date, standardized laboratory tests and well-defined parameters still need to be improved to predict the failure or success of therapy [27].

Given that bacteria growing in biofilms are more tolerant to antimicrobial agents than planktonic cells, effective combination therapies are necessary to eradicate biofilm-producing bacteria. In order to obtain more informative results, it is necessary to adapt the traditional cultivation techniques to the new challenges generated by the microorganisms present in PJIs [27]. De Vecchi et al. evaluated the role of DTT enriched with specific culture broths in optimizing the bacterial detachment, recovery, growth, and viability in diagnosing biofilm-related infections developed on orthopedic prosthetic devices. They demonstrated that DTT could be suitable for diagnosing biofilm-related infections [28]. Furthermore, Schoenmakers et al. used a conjugate of the antibiotic vancomycin and the near-infrared fluorophore IRDye800CW in combination with arthroscopic optical imaging to target and visualize biofilms on infected prostheses. The authors concluded that the presented image-guided arthroscopic approach provides direct visual diagnostic information and facilitates immediate appropriate treatment selection [29].

Linke et al. evaluated culture-positive synovial fluid samples of 192 consecutive patients; 132 suffered from PJI and 60 patients had infections of native joints. They aimed to analyze the impact of microbiological analysis in prescribing the appropriate antibiotics. They found coagulase-negative Staphylococci (28%), *Staphylococcus aureus* (26.7%), and other bacteria, such as streptococci (26.3%), as the most commonly isolated bacteria. They also found an increased detection rate of *Enterobacterales*. Detecting potentially causative bacteria raises the need to use early empiric antibiotic therapy [30]. The need for standardized antibiotic therapy for PJI treatment was stated in a study by Tsai et al., which analyzed the demographics and microbiological profiles of hip and knee PJI in 294 cases. The most common causative pathogen was *S. aureus* followed by coagulase-negative staphylococci, which had a high resistance rate to methicillin or oxacillin. These results may warrant empiric antibiotic therapy with broader coverage [31].

A big challenge in the microbiological diagnosis of PJI diagnosis is the presence of *C. acnes* in tissue samples, which for a long time was considered as a contaminant, especially if only found in a subset of the tissue specimens taken per patient. Although regarded as a low-virulence bacterium, it has been proposed that *C. acnes* may be a more common cause of PJIs than previously assumed and possibly sometimes misinterpreted as aseptic loosening [6,32]. However, there needs to be more consensus on strategies to prevent, diagnose, and treat postoperative shoulder infections. Standard surgical prophylactic regimens, such as intravenous antibiotics and topical chlorhexidine, are ineffective at removing *C. acnes* from the deep layer of the dermis and there is a shift toward using topical benzoyl peroxide with significantly improved efficacy. A deep understanding of this bacterium has demonstrated that a prolonged culture time of up to 14 days is needed, especially in cases of established infection. This bacterium is usually susceptible to a wide range of antibiotics, such as beta-lactams, fluoroquinolones, rifampin, and vancomycin. However, resistance is emerging to clindamycin [33].

### 2.2. Histopathological Diagnosis of PJIs

Histopathology of periprosthetic tissue is considered a standard procedure in PJI diagnosis. Histological analysis can be performed in parallel with the tissue culture analysis obtained during the arthroplasty surgery. It has been observed that the infection present in a patient is expressed differently in different anatomical areas, so biopsies from at least three sites are required [34]. Histopathological examination shows a high specificity and sensitivity in diagnosing PJIs. Based on histomorphological criteria, four types of periprosthetic lesions were defined: wear-particle induced, infectious, combined, and indeterminate type [35,36]. It has been observed that the nature of inflammatory infiltrate may vary even in the same patient, in different anatomical sites, which is why there is no unanimously accepted definition for acute infection. Usually, acute infection is identified by counting neutrophils and establishing histopathological scores [37]. Regarding the number of neutrophils existing in the inflammation level, the histological analysis suggests an infectious origin when identifying at least five neutrophils per site in at least five sites with 400X magnification [38], or, more recently, 23 neutrophils in 10 high-power fluorescence microscopy fields and more than 10 neutrophils in each high-power field [39].

Bemer et al. conducted a study on 215 patients to analyze this 23-neutrophil threshold and found that it could be proposed as a new histopathological gold standard for diagnosing PJIs [39]. Another study performed by Enz et al. confirmed the high value of histopathology in PJI diagnosis because of the same sensitivity and specificity compared to the microbiological analysis of the synovial sample. However, the authors concluded that the combined use of both methods could lead to a better increase in sensitivity by more than 18.0% and thus provide significantly more reliability for diagnosing low-grade infection. According to the results of this study, a preoperative histopathology sample should always be interpreted together with another synovial biomarker, and a microbiological culture and white blood cell count should be included in every synovial sample [40]. Tsaras and collaborators evaluated the role of the histopathological sections in diagnosing PJIs. They analyzed 26 studies with a total of 3269 patients, concluding that histopathological analysis of intraoperative prosthetic tissue is an essential part of PJI diagnosis, mainly if a complex infection assessment is performed before surgery [41].

Fernandez-Hijano performed a study including 133 hip and prosthetic knee replacements to assess the clinical validity of intraoperative histology in diagnosing PJIs. The histological analysis of the intraoperative samples showed high specificity, highlighting the crucial role of intraoperative diagnosis [42]. Another study by Padolino et al. compared sonication results with intraoperative tissue sample cultures. Among the 65 patients admitted in the study, tissue cultures were positive for the infection in 34 cases. In 19 cases, cultures were positive for *Cutibacterium acnes*. Sonication fluid cultures were positive in 40 cases (61.5%), with a positivity for *Cutibacterium acnes* in twenty-seven cases. The sensitivities of sonication and tissue cultures for the diagnosis of shoulder PJIs were 83.3% and 88.9%. These results suggest that the sonication technique had not shown a clear advantage in postoperative shoulder PJI diagnosis but could improve the detection of *Cutibacterium acnes*. In any case, sensitivity and specificity were higher with tissue cultures [43].

### 2.3. Imaging Diagnosis of PJIs

Concerning imaging diagnosis, conventional radiography is the most used for the initial diagnosis of PJIs. Examination of conventional radiographs may contribute to the early detection of asepticity loss. Although X-ray radiography can identify specific PJI features, such as osteolysis, subperiosteal elevation, and transcortical fistulas, its sensitivity and specificity in diagnosing infections are small, making it difficult to distinguish between loss of asepticity and infection in prosthetic joints [44]. Furthermore, conventional radiography may show demineralization only when more than 30–50% of bone mass has been lost, and abnormalities of bone around the implant are usually non-specific for infection [45].

Computed tomography (CT) offers a reasonable resolution of the bone and surrounding soft tissue, which is why it can be used in the pre-operative evaluation of bone defects [46]. Magnetic resonance imaging (MRI) can be used in patients who do not have ferrimagnetic implants. For knee arthroplasty, MRI is highly sensitive (92%) and specific (99%) for diagnosing PJIs. In addition, MRI offers better bone and soft tissue resolution than CT and plain radiography and does not involve ionizing radiation or contrast agents. The main disadvantage of CT and MRI techniques is the interference of the images near the metal implants [47].

Another fast, reliable, and high-quality imaging technique used for PJI detection is fluorine 18-fluorodeoxyglucose (FDG-PET) positron emission tomography [47]. Ultrasound may guide aspiration procedures of infectious materials in PJIs, evaluate periprosthetic fluid collections, and track the presence of sinus tracts within soft tissues. The main advantages of ultrasound are its wide availability, low cost, the possibility to perform it bedside, and repeated imaging without radiation burden [48].

Bone scintigraphy is usually performed after the injection of 99mTc-labelled diphosphonates, and three-phase bone scintigraphy can be performed to assess early perfusion, diffusion, and late bone uptake. The bone scintigraphy technique using the Tc isotope has excellent sensitivity in PJI diagnosis, but the specificity is low [49]. More recent studies have reported better results using immunoscintigraphy based on antigranulocyte antibodies in combination with labeled leukocytes and bone marrow scintigraphy [50]. Scintigraphy with Tc isotope-labeled monoclonal antibodies has a sensitivity of 83% and a specificity of 79% in PJI diagnosis. In comparison, scintigraphy with indium-labeled leukocytes in combination with bone marrow imaging presents a 90% accuracy in PJI detection [51].

Radiolabeled white blood cell (WBC) scintigraphy with both [99mTc] and [111In] can accurately differentiate a PJI from a mechanical aseptic loosening. However, it is well known that false-positive results may occur in physiologic bone marrow expansion because of migration of WBC in a reticuloendothelial system; therefore, a combination of bone marrow scintigraphy (BMS) and WBC scintigraphy is strongly suggested in order to improve accuracy [52]. Tomo-densitometry imaging (SPECT-CT) may help differentiate soft tissue infections from bone infections and identify the location of the lesion [14]. Blanc et al. conducted a study including 168 patients with mechanical joint loosening, aiming to evaluate the usefulness of leucocyte scintigraphy [LS] and SPECT-CT in relation to the pathogens involved in PJIs. A total of 150 patients underwent 99mTc-hexamethylpropyleneamine oxime (99mTc-HMPAO)-labeled leucocyte scintigraphy (LLS), and 18 underwent anti-granulocyte scintigraphy (AGS). Furthermore, 13 patients underwent additional single-photon emission computed tomography with tomodensitometry imaging (SPECT-CT). They demonstrated that LS is an exciting imaging modality to explore chronic bone and joint infections, providing helpful information in PJI diagnosis [53].

### 2.4. Molecular Diagnosis of PJIs

In recent years, molecular diagnostics positively impacted the process of identifying microorganisms. Molecular techniques can be used to detect pathogenic bacteria in cultures. Such techniques are polymerase chain reactions (PCR) and next-generation sequencing (NGS).

In PJI diagnosis, the PCR reaction can detect pathogens in the synovial fluid with a sensitivity of 84% and a specificity of 89%, and in the synovial fluid obtained by sonication, 81% and 96%, respectively [54]. More recently, it was observed that the sensitivity of the synovial fluid multiplex PCR overtakes thosethe one of tissue cultures; consequently, it is applied to the characterization of the bacterial colonies [55]. During recent years, several studies have shown the characteristics and usefulness of commercial multiplex PCR kits for diagnosing different bone and joint infections, such as SeptiFast by Roche, Genotype by Hain, Xpert by Cephaid, Filmarray by Biofire, and Unyvero i60 [56,57,58,59]. Along with multiplex PCR, a broad range of PCRs targeting the 16S gene have a sensitivity from 50% to 92% and a specificity from 65% to 94% [60]. The advantages of this technique are that the detection does not require cultivation, while the limitations refer to the risks of contamination that can lead to false-positive results [59].

Regarding NGS, the prospective study by Tarabichi and colleagues demonstrated the utility of this technique in detecting knee and hip PJIs, with a sensitivity of approximately 90%. The authors noted that NGS is more useful in detecting microorganisms in Gram-negative cultures and in detecting bacteria not identified in culture [61]. Street et al. evaluated the role of metagenomic sequencing in providing accurate diagnostic information in PJI diagnosis. They subjected 131 sonication fluid samples undergoing revision arthroplasty to metagenomic sequencing. Metagenomic sequencing, compared to the sonication fluid culture, provided a sensitivity of 88% and specificity of 88%. Sequencing was also able to detect potential pathogens not identified by the culture of sonication fluid. The authors identified additional species from sequencing that were supported by the tissue culture findings, suggesting that in some settings, sequencing may be more sensitive than sonication fluid culture alone [62]. Tan et al. conducted a meta-analysis including 10 new clinical studies to assess the diagnostic accuracy of metagenomic sequencing. The authors observed that metagenomic NGS had a high accuracy in PJI diagnostics, with a pooled sensitivity of 0.93 and a pooled specificity of 0.95, suggesting that it could be a novel approach for diagnosing clinical infectious diseases and address current pitfalls in clinical management [63].

NGS can provide auxiliary genomic information needed to predict drug resistance. In the case of PJIs involving more than one pathogen, NGS can also generate quantitative or semiquantitative data pertaining to bioconcentration by counting sequencing reads [64]. The main advantage of NGS is unbiased sampling, leading to the identification of known and novel organisms [65]. However, while metagenes can be used to detect pathogenic bacteria, they cannot be used for drug sensitivity tests simultaneously, which remains a persistent issue. Another disadvantage is that NGS is not feasible for routine testing because the price is still high at $500 or more per test [66].

## 3. Biomarkers

Biomarkers are a sub-category of medical signs that can be objectively measured and evaluated as an indicator of biological processes, pathogens, or pharmacological responses arising from therapeutic interventions. In practice, biomarker analysis involves using tools and technologies that help to understand the diagnosis, progression, and regression and the treatment needed [67]. In the case of patients with severe infections, biomarkers are broadly used to increase the correctness and the time of diagnosis, the early determination of the risk to provide a prognosis, and the prescription of personalized treatment correlated with the needs of each patient [68].

In the case of prosthetic infections, establishing a diagnosis is challenging because there is no standard test to confirm or exclude the infection. For this reason, a combination of tests is used in the clinic, each of which can be invasive and without absolute accuracy. Regarding biomarkers in the diagnosis of prosthetic infections, two major categories are used in the clinic: serum and synovial biomarkers.

### 3.1. Serum Biomarkers

Traditionally, serum biomarkers, such as ESR (erythrocyte sedimentation rate), CRP (C-reactive protein), interleukins, procalcitonin, and leukocyte counts, are used together with other tests to diagnose prosthetic infections [69]. For example, if there is a suspicion of a PJI, the CRP test is performed, which is a cheaper, faster, and more effective marker than ESR. However, many studies describe a broad range of sensitivities from 62% to 100% and specificities from 64% to 96% [70,71,72,73]. For example, Bottner and colleagues analyzed a series of serum markers (interleukin 6, procalcitonin, tumor necrosis factor, CRP, and ESR), noting that CRP and IL-6 have the highest specificity [74]. Similar results were obtained by Glehr et al., analyzing both preoperative and postoperative samples [75]. However, given the sensitivity and specificity of these serum markers, more is needed to diagnose or exclude PJI. Consequently, the clinical picture often is ambiguous, and although biomarkers, such C-reactive protein (CRP) or leukocyte levels, are helpful, they can be misleading in patients with chronic inflammatory diseases [75].

### 3.2. Synovial Biomarkers

Studies conducted in recent years have aimed at evaluating the efficiency of synovial biomarkers, which are a valuable tool in diagnosing prosthetic infections. Unlike serum biomarkers, which have low specificity because of their presence and other inflammation, synovial biomarkers have a high sensitivity and specificity because they are measured directly in the synovial fluid of the suspected joint [76].

The synovial biomarkers commonly include interleukins IL-6, IL-7, IL-17, TNF (tumor necrosis factor), and synovial CRP [77]. In addition, several more sensitive and specific synovial biomarkers have been evaluated in the diagnosis of PJI, such as alpha-defensin [78], cathelicidin LL-37, human beta-defensins 2 and 3 [79], leukocyte esterase [80], and calprotectin [81]. Although synovial CRP may be a practical diagnostic test [82], many laboratories prefer to measure CRP levels from synovial fluid because the devices can only be calibrated for serum CRP quantification.

Alpha-defensin is a synovial biomarker widely used in PJI diagnosis, which has a higher specificity than synovial CRP. The alpha-defensin test is an immune-type reaction that measures alpha-defensin concentration from the synovial fluid. Alpha-defensin is an antimicrobial peptide secreted into the synovial fluid by human cells in response to the presence of pathogenic bacteria [83]. It acts by integrating into the cell membrane of the pathogenic bacterium, leading to its rapid destruction, which is why it supports the antimicrobial activity of the immune system. In inflammatory conditions, the human beta-defensins 2 and 3 (HBD) secreted by neutrophils act against Gram-negative bacteria and *Candida*. Therefore, high levels of HBD-2 and HBD-3 were observed in patients diagnosed with PJIs [84].

Several studies support the role of alpha-defensins in PJI diagnosis. It has been observed that the alpha-defensin test provides remarkable results regardless of the type of organism, species, or virulence, so it can be considered a standard diagnostic method for PJIs [78]. The study by Bingham et al. shows that the alpha-defensin test in synovial fluid is more specific and sensitive than all other available clinical tests [82]. Kelly and co-workers analyzed 41 samples of synovial fluid obtained from patients who underwent a single arthroplasty operation and performed the alpha-defensin test to find its effectiveness in confirming the diagnosis of a PJI. Of the 23 samples in that antibiotic treatment was used, alpha-defensin confirmed the diagnosis for 19 samples (83%). It has also been observed that alpha-defensin from synovial fluid may support false positive or false negative results when PJI diagnosis cannot be transparent due to recent antibiotic treatment.

Given that only PJIs diagnosed, not suspected, were taken into account in this study, the sensitivity and specificity of this test demonstrate its usefulness in confirming and clarifying the PJI diagnosis [83]. All these studies confirm that alpha-defensin is a highly effective biomarker in diagnosing PJIs, providing essential information for clinicians. Another type of test used to detect alpha-defensin is ADFL (alpha-defensine lateral flow). Unlike the immune-based assay, ADFL is a qualitative test that determines the presence of alpha-defensin in the synovial fluid and can be performed during surgery or immediately after joint aspiration. Studies have shown that this test has a lower sensitivity when using sensitivity criteria for defining PJIs. However, it has a high specificity of approximately 99.3%, which is why it is still used for PJI detection [85].

Another biomarker used to diagnose PJI is leukocyte esterase (LE). LE is an enzyme secreted by activated neutrophils with high levels, especially in patients with different urological conditions. In the case of a PJI, neutrophils that reach the joint level following an infection produce LE that can be detected using colorimetric tests based on a color-changing reaction [86]. LE is a simple test that requires the application of synovial fluid on a strip used in urinary tests. Studies have shown that LE has a high specificity, positive predictive and negative value, and moderate sensitivity in PJI diagnosis [76]. Wetters and colleagues analyzed LE and reported a sensitivity between 92 100% and a specificity of approximately 90%. However, the LE test can be performed only in the case of blood-free samples because its presence may interfere with the color reaction of the colorimetric test [87].

LL-37 is an antimicrobial peptide from the cathelicidin family that induces the synthesis of immune mediators such as IL-8 [88], regulates the inflammatory response [89], and prevents biofilm formation [90]. Gallwitzer et al. observed high levels of LL-37 in synovial fluid collected from PJI patients and reported a specificity of 85% in PJI diagnosis [79].

Another biomarker analyzed in PJI is calprotectin. The calprotectin identification test is an immune reaction that measures calprotectin concentration in the synovial fluid [91]. It is a heterodimeric complex composed of two myeloid proteins. This protein is a pro-inflammatory factor of innate immunity that activates toll-like receptor (TLR) four and is released by activated granulocytes during the inflammatory process [92]. The first study that evaluated calprotectin concentration in synovial fluid was by Wouthuyzen-Bakker et al. In this study, it has been shown that measuring calprotectin levels has particular importance in establishing the diagnosis of PJIs [81]. Subsequently, Salari and colleagues analyzed 76 patients with painful knee arthroplasty to observe the association of calprotectin with the infectious process. Calcprotectin analysis of synovial fluid showed that this test has a sensitivity of 100% and a specificity of 95% in PJI diagnosis. It was also observed that the average concentration of calprotectin in the samples from patients with infection was 58-fold higher than the level of calprotectin in the aseptic samples [93]. The same research group conducted a more recent prospective observational study involving 93 patients suffering from painful PJI and compared the sensibility and sensitivity of both the enzyme-linked immunosorbent assay (ELISA) calprotectin test and the rapid calprotectin test (CalFAST) to leukocyte esterase (LE). These authors demonstrated that the calprotectin ELISA test and CalFAST had a similar sensitivity (92.3% and 97.4%, respectively) and specificity, whereas the LE rapid test showed 46% sensitivity and 94% specificity. The authors concluded that synovial CPT has high accuracy in knee PJI diagnosis, and both ELISA and rapid tests are valid. Consequently, the CPT rapid test can be considered an excellent point-of-care test in clinical practice [94]. These studies show that measuring the concentration of calprotectin in the synovial fluid has high sensitivity and specificity in establishing the diagnosis of PJIs.

### 3.3. Emerging Biomarkers in PJI Diagnosis

Given the connections between the coagulation cascade and bacterial inflammatory mechanisms [95], coagulation regulators, such as infectious biomarkers, may play an essential role in prosthetic infections diagnosis. Fibrinogen, a precursor of fibrin, is a glycoprotein that plays a vital role in the coagulation cascade and in activating and mediating the inflammatory process by inducing the synthesis of proinflammatory cytokines [96]. Given these mechanisms, the hypothesis of using fibrinogen as a biomarker in PJI diagnosis has been raised. Alturfan et al. observed that fibrinogen is an essential parameter in arthroplasty infection with a sensitivity of 93% and a specificity of 86% [97], while Sedlar et al. showed that fibrinogen has high levels in PJIs prior to surgery [98]. Recent studies have confirmed the sensitivity and specificity of fibrinogen in PJI detection, obtaining comparable values with other serum biomarkers (CRPs) in terms of diagnostic accuracy [99]. However, further research is needed to validate these studies and to identify other infection biomarkers.

Deirmengian and co-workers analyzed synovial fluid samples from 95 patients and tested 16 biomarkers to observe their predictive degree in PJI diagnosis. Of the 16 biomarkers tested, 5 biomarkers (alpha-defensins 1–3, elastase two from neutrophils, bacterial/permeability-increasing protein, lipocalin associated with neutrophils, and lactoferrin) had a sensitivity and specificity of 100% in PJI diagnosis [100]. Regarding lipocalin, the study by Li and colleagues confirmed the results obtained previously, demonstrating that lipocalin 2 is a potential biomarker with an essential value in diagnosing PJIs. Lipocalin showed elevated concentrations in patients with inflammatory joint disease; however, more comprehensive studies are needed before introducing lipocalin as a standard diagnostic tool [101].

Presepsin (sCD14-ST) is a subtype of soluble CD14 found on the surface of monocytes during the inflammatory response and is released into the blood, serving as a marker for infection [102]. It can also bind to peptidoglycan and other molecules found in Gram-negative or Gram-positive bacteria as it is involved in the immune response [103]. Considering the mechanisms involved, recent studies have examined the possibility of using presepsin as a biomarker in PJI diagnosis. Imagama et al. conducted a study to evaluate synovial fluid, serum presepsin, and procalcitonin levels in 18 patients with septic arthritis compared with 28 patients affected by osteoarthritis to determine whether presepsin would be helpful in the diagnosis of septic arthritis. Synovial fluid presepsin exhibited both 100% sensitivity and 100% specificity in the septic arthritis group at higher rates than those for blood presepsin and procalcitonin. Thus, Imagama et al. concluded that synovial fluid presepsin could be a new biomarker of septic arthritis [104]. However, a more recent study has limited the diagnostic and prognostic role of synovial fluid PS in PJIs compared to serum presepsin concentration; thus, future studies with larger samples are needed to more accurately describe the role of synovial presepsin concentration in PJI diagnosis [105].

Another potential biomarker in PJI diagnosis is chemokine ligand 2 (CCL2), a protein produced by monocytes and dendritic cells. Galliera and co-workers observed that CCL2 values were higher in patients with PJI than in the control group. Furthermore, CCL2 levels were observed to be proportional to those of the serum suPAR receptor, which underlines the potential of CCL2 as a biomarker in PJI diagnosis [106].

In addition to the biomarkers presented above, recent studies have presented several potential novel biomarkers for PJI diagnosis, such as TLR 2, soluble urokinase plasminogen receptor (suPAR), osteopontin, and enriched cysteine with EGF-like domains (CRELD2). TLR 2 is usually found on the surface of antigen-presenting cells and plays an essential role in triggering the innate immune response. Galliera and co-workers demonstrated the involvement of these receptors in PJIs, where they play a role in the detection and response to Gram-positive bacteria [107]. Marrazi and co-workers measured their concentration in synovial fluid samples, observing a concentration lift that decreased slightly within the first 48 h after the arthroplasty [108]. suPAR is found in two forms, a membrane-bound form (uPAR) and a soluble form (suPAR). suPAR plays a vital role in immune functions, such as cell migration and adhesion, chemotaxis, immune activation, and invasion, and is found in blood and other fluids such as synovial fluid [109]. The role of suPAR in PJIs was recently highlighted by Galliera and colleagues in a study of 80 patients with PJIs. It has been suggested that this marker could contribute to the PJI diagnosis, having even greater sensitivity and specificity than CRP and IL-6 [106].

Although the performed studies offer essential results regarding the use of these new potential biomarkers, further studies are needed on larger groups of patients so they can be validated in diagnosing PJIs.

## 4. Common Errors in PJI Diagnosis

Despite the increased interest in diagnosing and managing PJIs, recent meta-analyses have reported microbiological failure rates of 0–40% for one- and two-stage revisions for infected hip and knee arthroplasties [110]. Failure may result from patient and microbiological factors or other causes related to errors during diagnosis and treatment.

One of the most critical challenges in PJI treatment is delayed diagnosis, which can lead to a decrease in the effectiveness of debridement, antibiotics, and implant retention (DAIR). The most influential determinants of DAIR outcome are the timing of debridement from the onset of symptoms and the exchange of modular components at the time of the initial debridement and, to a lesser extent, the time from the initial procedure [111]. A retrospective review by Zhang et al. that included 24 patients showed that DAIR has a high success rate for treating acute PJIs and may be performed in selected patients whose symptoms have been sustained for over four weeks. Furthermore, a high success rate was reported for using DAIR for staphylococcal infections [112]. In a severe case of PJI investigated by Lucero et al., it was observed that the chances of successful treatment increase with an accurate and early diagnosis involving multidisciplinary management that includes clinicians, an infectionist, and hip surgeons [113].

Another factor that could negatively influence the PJI diagnosis is using tissue swabs in microbiological culture samples. Previous reports have shown that the sensitivity of swab culture is low (53–76%) and is often associated with the misidentification of causative pathogens [114]. Results of swab-based samples from draining wounds or sinus tracts are misleading as they could produce polymicrobial or false-positive results due to contamination with skin flora, such as coagulase-negative staphylococci and *Cutibacterium* spp. [115]. Considering that accurate microbial diagnosis is crucial for effectively managing PJIs, Larsen et al. proposed a new concept of pre-packed boxes that include swabs, vials, and additional tools needed in the operating theatre for non-standard samples. This protocol requires triplicate samples of joint fluid, periprosthetic tissue, bone tissue, and swabs from the surface of the prosthesis and has been evaluated in 164 cases of surgery [116].

Another possible error in PJI diagnosis is using CRP and ESR as first-line tests in suspected PJIs because of their convenience and short waiting times. However, many studies show a broad range of sensitivities from 62% to 100% and specificities from 64% to 96% [70,71,72,73]; none is sufficient to diagnose or exclude PJIs. Furthermore, the 2018 Proceedings of International Consensus Meeting (ICM) on Orthopedic Infections underlined that negative test results do not exclude the possibility of infection [117].

Tissue sampling culture is the most commonly used intra-operative diagnostic method. The Infectious Diseases Society of America recommends collecting at least three and, optimally, five or six periprosthetic intra-operative tissue samples for aerobic and anaerobic culture [118]. However, a study conducted by Peel et al. suggests that the most remarkable accuracy is obtained with three periprosthetic tissue samples cultured compared to the five to six recommended in current guidelines [119]. Tissue sampling methods are critical in PJI diagnosis due to the risk of false-positive or false-negative results. First, tissue samples should be obtained using sharp dissection, avoiding electrocautery to limit false-positive results due to thermal artifacts that could appear in the histopathologic analysis [120]. Second, samples should be collected from the areas where signs of infection are more pronounced and from different areas of the surgical field (e.g., in the femoral canal, hip revisions, and the bottom of the acetabulum) [121]. Third, surgical instruments should be changed for each tissue sample to avoid a risk of cross-contamination between samples, which could impact culture results [122]. Fourth, sonication of the removed implants in polyethylene bags increases the risk of microbial contamination leading to a false-positive result [19].

Another standard error in PJI treatment is the lack of personalized therapies. Treatment of an infected prosthesis should be designed according to the type of infection, causative microorganism, the quality of the soft tissue envelope, stability of the implant, surgeon experience, comorbidities and functional status, and patient preferences [1]. The prosthesis must be removed when chronic PJI is associated with a mature biofilm. This operation can be performed in one or two stages, depending on the causative microorganisms, soft tissue condition, and surgeon and patient preference. In the USA, a two-stage exchange of an infected prosthesis is considered the gold standard and is the dominant option, and in some European countries, a single-stage exchange is favored. However, a one-stage exchange may not be an option in patients with signs of systemic sepsis, extensive comorbidities, and infection with resistant organisms [123]. Antibiotic treatment strategies should be tailored to the type of microorganism, drug susceptibility, and surgery performed. When surgeons are dealing with sessile bacteria embedded in biofilm (e.g., *Staphylococcus* species, *Cutibacterium* species, Gram-negative rods), they should remember that not all antibiotics are equally active, and these should be reserved for the period after implantation of the definitive implant. Another error is prescribing oral antibiotics with low bone penetration and poor oral bioavailability, resulting in insufficient local concentrations at the site of infection (e.g., beta-lactam antibiotics) [124,125]. Since choosing the correct antibiotic cocktail is complex, an orthopedic surgeon should not make the decision alone. Infectious disease specialists with experience treating implant infections must work closely with the surgeon and should understand its impact and importance to the patient.

## 5. Conclusions

Preoperative diagnosis of PJI is crucial given the therapeutic consequences, yet it remains challenging due to the variety of clinical symptoms and unclear significance of systemic biomarkers. Therefore, special attention should be given to new serum and synovial biomarkers that will likely play a critical role in the screening for PJI in coming years. In addition, research and development of new diagnostic methods with more accuracy, simplicity, and convenience will help improve our ability to diagnose PJIs quickly and avoid possible devastating outcomes. To achieve this desideratum, interdisciplinary teams are needed and should include, at a minimum, microbiologists, infectious disease specialists, and orthopedic and plastic surgeons.
